# The complete mitochondrial genome of *Cynoglossus interruptus* and its novel rearrangement (Pleuronectiformes: Cynoglossidae)

**DOI:** 10.1080/23802359.2019.1660262

**Published:** 2019-09-06

**Authors:** Ha Yeun Song, Jin-Koo Kim, Seonmi Jo, Seung-Hyun Jung, Bora Kim, Young Ji Choi, Jong Su Yoo, Dae-Sung Lee

**Affiliations:** a Department of Genetic Resources Research, National Marine Biodiversity Institute of Korea, Seocheon-gun, Republic of Korea;; b Department of Marine Biology, Pukyong National University, Busan, 48513, Republic of Korea

**Keywords:** Mitochondrial genome, Pleuronectiformes, Cynoglossidae, *Cynoglossus interruptus*

## Abstract

The complete mitochondrial genome was determined for the *Cynoglossus interruptus* belonging to the family Cynoglossidae. The length of the complete mitochondrial genome is 17,262 bp, consisting of 13 protein-coding genes, 22 tRNA genes, two rRNA genes, and a control region. The gene rearrangement related to tRNA^Gln^ and a control region gene were found, forming the gene order of CR-Ile-Gln-Met. Phylogenetic analysis using mitochondrial genomes of 12 species showed that *C. interruptus* formed a well-supported monophyletic group with other *Cynoglossus* species.

The Genus *Cynoglossus* (Pleuronectiformes: Cynoglossidae), including about 50 species, is distributed in tropical and subtropical waters from the Atlantic coast of Africa to Western Pacific (Menon 1977). In the Genus *Cynoglossus*, six species comprising *C. abbreviates*, *C. gracilis*, *C. semilaevis*, *C. joyneri*, *C. robustus*, and *C. interruptus* were recorded from Korea (Jordan and Metz 1913; Mori 1928; Mori and Uchida 1934; Uchida and Yabe 1939; Mori 1952). In previous studies, Yokogawa et al. (2008) reported that confirmation regarding the distribution of the *C. interruptus* among six species in Korean waters is required, and Kwun and Kim (2016) have re-identified that the *C. interruptus* is distributed in Korean waters using morphological and molecular data. In this study, we first determined the complete mitochondrial genome of *C. interruptus*.

The *C. interruptus* specimen was collected from Sacheon-si, Gyeongsangnam-do, Republic of Korea (34.56 N, 127.58E). Total genomic DNA was extracted from tissue of the specimen, which has been deposited in the Marine Fish Resources Bank of Korea (MFRBK) (Voucher No. PKU56300). The mitogenome was sequenced and assembled using Illumina Hiseq 4000 sequencing platform (Illumina, San Diego, CA) and *SOAP denovo* assembler at Macrogen Inc. (Korea), respectively. The complete mitochondrial genome was annotated using MacClade ver. 4.08 (Maddison and Maddison 2005) and tRNAscan-SE 2.0 (Lowe and Chan 2016).

The mitochondrial genome of *C. interruptus* (GenBank accession no. LC482306) is 17,262 bp long, including 13 protein-coding genes, 22 tRNA genes, two rRNA genes, and a control region. The overall base composition is 30.53% A, 24.38% C, 14.98% G, and 30.11% T, with a bias on AT content (60.64%). The 12S rRNA (943 bp) and 16S rRNA genes (1,685 bp) are located between tRNA^Phe^ and tRNA^Val^ and between tRNA^Val^ and tRNA^Leu(UUR)^, respectively. Of the 13 protein-coding genes, 11 begin with an ATG start codon; the exception being the *COI* gene and *ND3*, which start with GTG and ATT, respectively. The stop codon of the protein-coding genes is TAA in *ND1, COI*, *ATP8*, *ND4L, ND5*, *ND6* and *Cytb*; TAG in *ND2*; TA– in *ATP6* and *COIII*; and T–– in the remaining three genes.

Additionally, gene rearrangements related to tRNA-Gln gene and control region were found. The tRNA-Gln gene has translocated from the light to the heavy strand and the control region translocated downstream to the place between ND1 and tRNA-Ile genes. Also, we detected unique gene order, CR-Ile-Gln-Met, which is different from other *Cynoglossus* species, forming the gene order of CR-Gln-Ile-Met (Kong et al. 2009; Shi et al. 2015; Wei et al. 2016; Bo et al. 2017; Chen et al. 2019).

Phylogenetic trees were constructed by the maximum-likelihood method using MEGA 7.0 software (Kumar et al. 2016) for the newly sequenced genome and a further 11 mitochondrial genome sequences downloaded from the National Center for Biotechnology Information. We confirmed that *C. interruptus* formed a monophyletic group with other *Cynoglossus* species (Figure 1). The mitogenome features of the *C. interruptus* provide important molecular data to confirm that *C. interruptus* is distributed in Korean waters.

**Figure 1.**
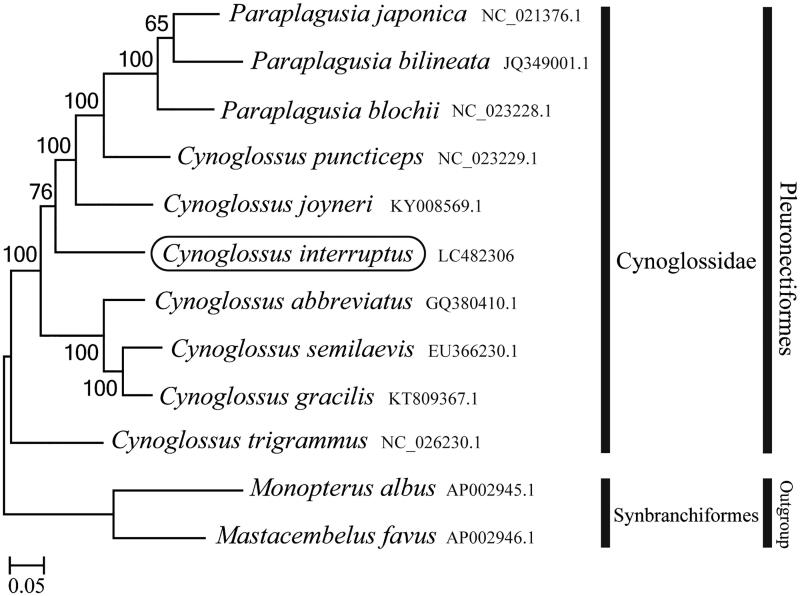
Phylogenetic position of *Cynoglossus interruptus* based on a comparison with the complete mitochondrial genome sequences of 12 species. The analysis was performed using MEGA 7.0 software. The accession number for each species is indicated after the scientific name.
